# Unbalanced Glutamine Partitioning between CD8T Cells and Cancer Cells Accompanied by Immune Cell Dysfunction in Hepatocellular Carcinoma

**DOI:** 10.3390/cells11233924

**Published:** 2022-12-04

**Authors:** Jianfei Chen, Rui Wang, Zhongliang Liu, Jun Fan, Shenglu Liu, Shunde Tan, Xinkai Li, Bo Li, Xiaoli Yang

**Affiliations:** 1 Department of General Surgery (Hepatobiliary Surgery), The Affiliated Hospital of Southwest Medical University, Luzhou 646000, China; 2 Academician (Expert) Workstation of Sichuan Province, Luzhou 646000, China; 3 Nuclear Medicine and Molecular Imaging Key Laboratory of Sichuan Province, Luzhou 646000, China

**Keywords:** primary hepatocellular carcinoma, glutamine metabolism, CD8T cell subpopulation, immunotherapy, JHU083

## Abstract

Glutamine metabolism is critical both for the proliferation of cancer cells and the activation of CD8T cells to kill cancer cells. We aim to explore the relationship between the glutamine metabolism of CD8T cells and cancer cells and tumor immunity in the tumor microenvironment. In a TCGA cohort, we found that patients with high scores of glutamine-metabolism-related genes showed poor prognoses, and that a high score of glutamine-metabolism-related genes was an independent risk factor for HCC patients. In single-cell RNA-seq data, we found that, in some patients, the glutamine metabolism gene scores of tumor cells were significantly higher than those of CD8T cells, while decreased ratios of CD8-Tef-GZMA and suppressed tumor-killing activity of CD8-Tef-APOC2 were observed. A further genetic dynamics pseudotime analysis suggested that immune remodeling of these two subpopulations was accompanied by metabolic reprogramming. CD8-Tef-APOC2 in the dominant group tended to metabolize exogenous lipids, while the metabolic program of CD8-Tef-GZMA in the nondominant group was characterized by amino acid and endogenous lipid synthesis. In addition, we found that the glutamine metabolism inhibitor JHU083 promoted the proliferation of CD8T cells and improved the efficacy of PD-1 blockers. We proposed a new tool to quantify the glutamine partitioning between tumor cells and CD8T cells, through which the unique immune microenvironment could be identified at the transcriptome level. Furthermore, the simultaneous destruction of the glutamine metabolism in tumor cells and CD8T cells facilitated the enrichment of tumor-infiltrating CD8T cells and enhanced the efficacy of immunotherapy.

## 1. Introduction

The CD8T subpopulation are critical lymphocytes with antitumor effects on the immune microenvironment of hepatocellular carcinoma (HCC). They produce perforin and other cytotoxins, killing cancer cells without damaging normal cells. Existing studies have shown that the immune lethality of the CD8T subpopulation is closely related to immune phenotype and abundance. As early as 2009, Prof. Thommen systematically summarized the characteristics of sugar depletion and the lipid metabolism reprogramming of T cells in a low-glucose, hypoxic and high-lactate tumor microenvironment [1]. Previous classical research has considered the high glucose metabolism of tumor cells as the core factor that reshapes the metabolic microenvironment and prevents CD8T cells from exerting their antitumor ability [2]. Unfortunately, a large number of drugs targeting the glucose metabolism in tumor cells have failed to achieve clinical translation [3]. In 2021, Rathmell et al. analyzed the metabolic characteristics of various cells in the microenvironment of colon cancer with nucleotide-tracing techniques and found for the first time that CD8T cells did not lack glucose; cancer cells, in contrast, presented a glutamine uptake four-fold higher than that of CD8T cells. Therefore, perhaps it is the unique nutrient partitioning of glutamine in the immune microenvironment that affects the antitumor immunity of CD8T cell subsets [4].

Activated immune cells require unique metabolic patterns to meet their enhanced energy and biosynthetic needs (amino acids, nucleotides, lipids, etc.) Glutamine is responsible for mitochondrial anaplerosis, nucleotide synthesis, amino acid production, and redox homeostasis during immunophenotypic remodeling, and is known as “immune fuel” [5]. For example, insufficient intake of glutamine inhibited the proliferation and cytokine production in T lymphocytes [6]; similarly, the knockdown of glutaminase (GLS) blocked the conversion of glutamine to glutamate and reduced the response to immunotherapy [7]. On the other hand, glutamine utilization in malignant cells is recognized as a characteristic of cancer. For instance, loss of the proliferative activity of breast cancer cells was observed in glutamine-depleted cultures [7]. Suppressing the expression of the high mobility group box 1 (HMGB1) gene could reduce glutamine metabolic activity in hepatocellular carcinoma cells and significantly enhance the response of HCC cells to PD-L1 antagonists [8]. This evidence suggests that both tumor cells and immune cells in the immune microenvironment are glutamine-dependent, which implies that we have grossly underestimated the role of glutamine metabolism in shaping the different immunophenotypes of tumor-infiltrating CD8T cells.

Therefore, we speculate that the tumor-killing ability of the CD8T subpopulation can be inhibited when the glutamine metabolic capacity of tumor cells is much higher than that of CD8T cells. In addition, disrupting this abnormal tumor metabolic microenvironment may improve CD8T cell function and contribute to the efficacy of immunotherapy.

## 2. Results

### 2.1. Patients with High Glutamine Metabolism-Related Gene Expression Score Suffer Worse Prognoses in TCGA Cohort

**Figure 1:**
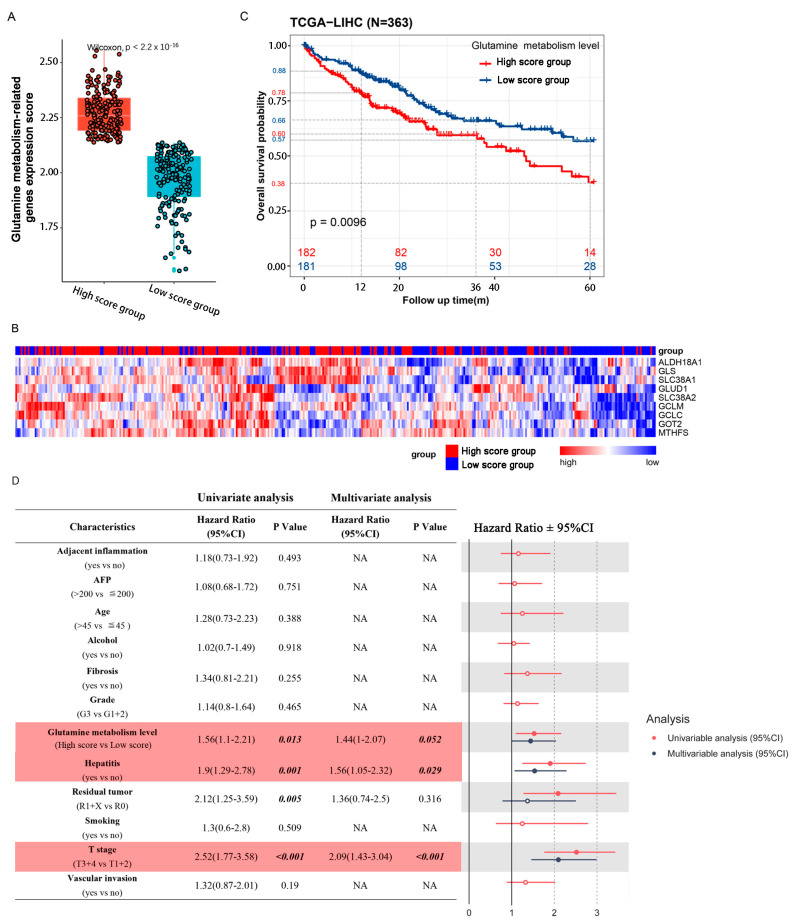
Glutamine metabolic activity of tumor tissue is associated with patient prognosis. (**A**) 363 patients of a TCGA cohort could be divided into 2 groups according to the median value of glutamine-metabolism-related gene expression scores. (**B**) Expression levels of individual genes (ALDH18A1, GCLC, GCLM, GLS, GLUD1, GOT2, MTHFS, SLC38A1 and SLC38A2) are displayed as a heatmap, with low expressions in blue and high expressions in red. The left side of the heatmap shows patients with high scores and the right side shows patients with low scores. (**C**) Kaplan–Meier analysis shows that patients with high scores suffered a worse overall survival rate. Numbers of patients and risk classifications are indicated in the figure. Significance was calculated using the log-rank test. (**D**) CPHR analysis results of glutamine-metabolism-related gene expression scores and clinical factors. The forest on the right shows the results of the univariate and multivariate analyses, with reference value of the hazard ratio = 1. Line intersection with reference line suggests no statistical difference. Red represents univariate analysis, and blue represents multivariate analysis.

We calculated the cancer cell glutamine-metabolism-related gene expression scores of 363 patients in a TCGA cohort, and divided them into a high score group and a low score group according to the median value (Figure 1A). Hierarchical clustering was also performed for all the glutamine-metabolism-related genes, and we found that the 363 HCC patients could be separated by unsupervised statistical techniques. Although some samples overlapped with other groups, there was good separation between the high and low score groups (Figure 1B). A Kaplan–Meier plot showed shorter overall survival times in HCC patients with high scores. In the high score group, the survival rates were 78% at 1 year, 60% at 3 years, and 38% at 5 years, while the 1-year, 3-year, and 5-year survival rates of the low score group were 88%, 66%, and 57%, respectively (Figure 1C). We evaluated associations between 12 clinical pathological parameters and prognosis in both univariate and multivariable models. In the univariate analysis, residual tumor (HR = 2.12; *p* = 0.005), history of hepatitis (HR = 1.9; *p* = 0.001), T-stage (HR = 2.52; *p* < 0.001) and glutamine-metabolism-related gene expression scores (HR = 1.56; *p* = 0.013) were significantly associated with increased death risk. After the multivariable analysis, only glutamine-metabolism-related gene expression scores (HR = 1.44; *p* = 0.052), history of hepatitis (HR = 1.56; *p* = 0.029) and T-stage (HR = 2.09; *p* < 0.001) were significantly associated with increased death risk and were defined as independent risk factors for OS (Figure 1D and Table S3).

### 2.2. Patient Grouping and Subpopulation Annotation of Single-Cell Sequencing Data

**Figure 2:**
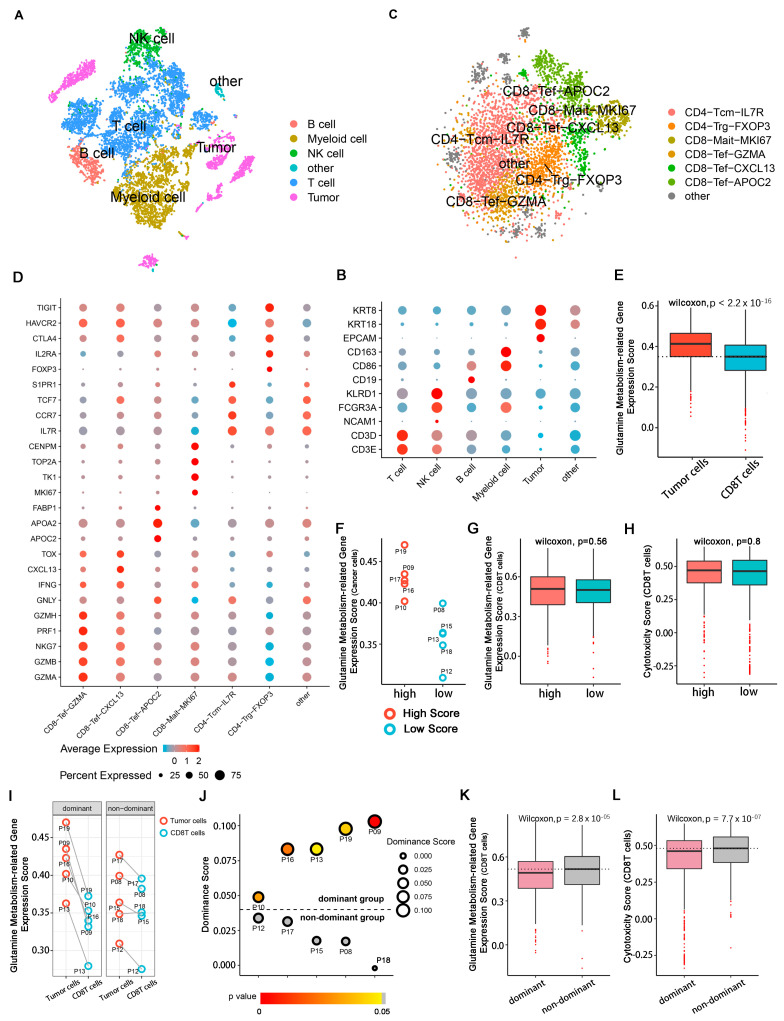
Cell annotation and HCC patient grouping in single-cell RNA-seq. The single-cell RNA sequencing data were downloaded from the China National GeneBank (https://db.cngb.org/search/project/CNP0000650/ (accessed on 15 March 2022). (**A**) t-SNE plot showing the cell subpopulations in the TME derived from HCC patients. Each cluster is color-coded according to cell type. Cluster annotations are indicated in the figure. (**B**) Dot plot showing the expressions of marker genes in each cell subpopulation. The colors of the dots indicate the average expression level: red shows high expressions and blue shows low expressions. Sizes of the dots represent cell proportions. (**C**) t-SNE plot showing the cell subtypes derived from T cells. (**D**) Dot plot showing the expressions of marker genes in each subpopulation of T cells. (**E**) Box plot showing the differences in glutamine-metabolism-related gene expression scores between tumor cells and CD8T cells. (**F**) Scatter plot of cancer cell glutamine-metabolism-related gene expression scores in 10 HCC patients. Red points represent HCC patients with high scores; blue points represent patients with low scores. (**G**,**H**) Box plots illustrating the differences in glutamine-metabolism-related gene expression scores and cytotoxicity scores in CD8T cells among different patients. (**I**) Scatter plot showing the differences in glutamine-metabolism-related gene expression scores between cancer cells and CD8T cells in each patient. Information about patients is listed in Supplementary Table S4. (**J**) Volcano dot plot showing metabolic classifications of 10 patients. The color of each dot indicates the *p*-value. Gray indicates no difference between cancer cells and immune cells in a patient. Dominance score is represented by dot size. Patient ID is marked in the figure. (**K**–**L**) Box plots illustrating the differences in glutamine-metabolism-related gene expression scores and cytotoxicity scores of CD8T cells between dominant and nondominant groups.

To obtain a complete single-cell expression atlas in the TME of primary liver cancer, cell classification and marker gene identification were performed with Seurat based on 10 qualified primary hepatocarcinoma patients. Five main cell clusters and an undefined cluster were defined for 8927 eligible cells by the T-distributed stochastic neighbor embedding (t-SNE) method (Figure 2A,B and Figure S2). The main cell clusters included T cells (CD3E and CD3D), B cells (CD19), natural killer cells (NCAM1, FCGR3A and KLRD1), myeloid cells (CD86 and CD163) and tumor cells (EPCAM, KRT8 and KRT18). t-SNE plots of each marker gene are shown in Supplementary Figure S3. The undefined group without known biological markers was not studied in our research.

The total amount of 4773 T cells was further divided into six known subpopulations, as well as another undefined subpopulation, based on known molecular markers after the removal batch effects, dimensionality reduction, and re-clustering (Figure 2C,D and Figure S2). The CD4+ T subpopulation included central memory CD4 IL7R^high^ T cells (CD4-Tcm-IL7R: IL7R, CCR7, TCF7 and S1PR1) and classical regulatory CD4 FXOP3^high^ T cells (CD4-Trg-FXOP3: FXOP3, IL2R, CTLA4, HAVCR2 and TIGIT). The CD8+ cluster included proliferating CD8 MKI67^high^ T cells (CD8-Mait-MKI67: CENPM, TOP2A, TK1 and MKI67) and three subpopulations of CD8 effector T cells, including GZMA^high^ T cells (CD8-Tef-GZMA: GZMA, GZMB, GZMH, PRF1, NKG7 and IFNG), CXCL13^high^ T cells (CD8-Tef-CXCL13: GZMA, GZMB, GZMH, PRF1, NKG7, IFNG, CXCL13 and TOX), and APOC2^high^ T cells (CD8-Tef-APOC2: GZMB, GZMH, NKG7, GNLY, APOA2, APOC2 andFABP1). t-SNE plots of each marker gene are shown in Supplementary Figure S4. Some of the T cells could not be identified by known molecular markers after clustering and were not biologically annotated here.

We extracted glutamine-metabolism-related genes in cancer cells and CD8T cells from each patient and separately calculated the expression scores of these genes. We found that the glutamine-metabolism-related gene expression scores of cancer cells were significantly higher than those of the CD8T cells (Figure 2E). Further, we attempted to explore whether cancer cells with high expression scores of glutamine-metabolism-related genes affected the glutamine metabolism and tumoricidal abilities of CD8T cells. We divided the 10 patients into a high score group (P09, P10, P16, P17 and P19) and a low score group (P8, P12, P13, P15 and P18) according to the glutamine-metabolism-related gene expression scores of cancer cells (Figure 2F). Unfortunately, we did not observe significant changes in glutamine-metabolism-related gene expression scores or cytotoxic scores of CD8T cells between the two groups (Figure 2G,H). However, we observed a significant difference in expression scores for glutamine-metabolism-related genes between cancer cells and CD8T cells among some patients (Figure 2I), so we divided the patients into different groups with distinct cytotoxic scores for CD8T cells. According to the expression score difference between these two cell subpopulations, patients were divided into the cancer cell groups of glutamine-metabolism-related gene dominant expression scores (dominant group) and nondominant expression scores (nondominant group). The difference in glutamine-metabolism-related gene expression scores between those two subpopulations was defined as a dominant score. Five patients (P09, P10, P13, P16 and P19) could be classified in the dominant group (dominance score > 0 & *p* < 0.05) and the other five patients (P08, P12, P15, P17 and P18) were in the nondominant group (dominance score < 0 or *p* > 0.05) (Figure 2J). In the dominant group, we observed that the glutamine-metabolism-related gene expression scores of CD8T cells were significantly downregulated (Wilcoxon test, *p* = 2.8 × 10^−5^) (Figure 2K), accompanied by a decrease in cytotoxic score (Wilcoxon test, *p* = 7.7 × 10^−7^) (Figure 2L). This may indicate that it was the glutamine metabolism dominance score difference between cancer cells and CD8T cells, rather than cancer cell glutamine-metabolism-related gene expression scores, that affected the tumoricidal ability of CD8T cells.

### 2.3. Tumoricidal Activity of CD8T Cells Is Suppressed in the Dominant Group

**Figure 3:**
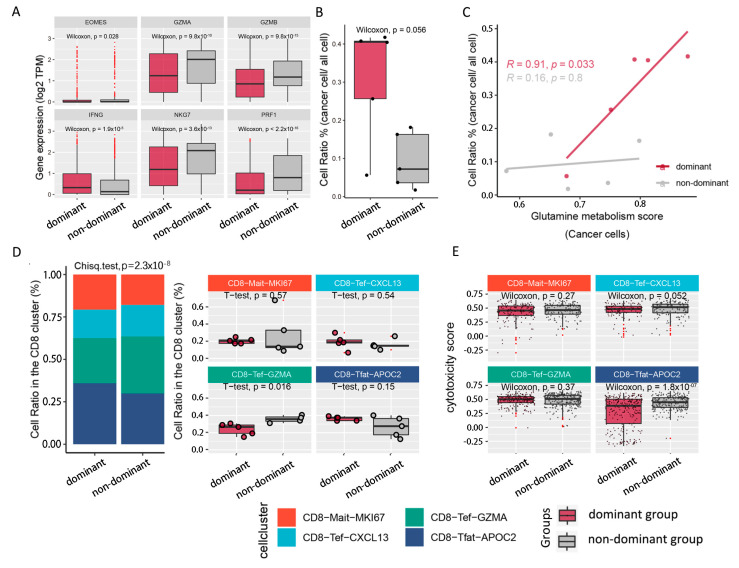
Differences in composition ratios and antitumor abilities in CD8T subpopulations. (**A**) Box plot showing the expressions of cytotoxicity-related genes (GZMA, GZMB, PRF1, IFNG, EOMES and NKG7). (**B**) Box plot illustrating cancer cell ratios in the TME of different populations. (**C**) Fitting curve presenting the correlation between glutamine-metabolism-related gene expression scores and cell ratios of cancer cells; correlation coefficients and significance tests are annotated in the figure. Gray indicates nondominant group, and light purple indicates dominant group in (**A**–**C**). (**D**) Stacked bar plot on the left shows the cell ratios of CD8T cell subtypes in the TME of dominant group vs. nondominant group. Box plot on the right compares the cells ratios of CD8T cell subtypes between the 2 populations. (**E**) Box plot representing the difference in cytotoxicity scores in CD8T cell subtypes between 2 populations. Metadata of CD8T cells in the TME of 10 primary hepatocellular carcinoma patients are listed in Supplementary Table S5.

We further compared the cytotoxic gene expression levels (EOMES, GZMA, GZMB, IFNG, NKG7 and PRF1) of CD8T cells between the two groups, and it was observed that the expressions of these classical cytotoxic genes were significantly downregulated in the dominant group (Figure 3A), especially for GZMA (Wilcoxon test, *p* = 9.8 × 10^−10^), GZMB (Wilcoxon test, *p* = 9.8 × 10^−15^), NKG7 (Wilcoxon test, *p* = 3.6 × 10^−13^) and PRF1 (Wilcoxon test, *p* = 2.2 × 10^−16^). Next, we compared the composition ratios of cancer cells in the two populations. In the dominant group, a higher proportion of cancer cells was observed (Figure 3B), which increased with the enhancement of cancer cell glutamine-metabolism-related gene expression scores (correlation coefficient R = 0.91; *p* = 0.033) (Figure 3C). To evaluate the tumoricidal capacity in the CD8T subpopulations, we calculated the cell ratios and cytotoxicity scores in four CD8Tef subpopulations. Interestingly, we observed decreased CD8-Tef-GZMA (*t*-test, *p* = 0.016) in the dominant group, while the proportions of CD8-Tef-APOC2, CD8-Tef-CXCL13 and CD8-Mait-MKI67 cells were increased without significant change (Figure 3D). At the same time, the cytotoxicity scores of CD8-Tef-APOC2 (Wilcoxon test, *p* = 1.8 × 10^−7^) in the dominant group were weaker, while the cytotoxicity scores of CD8-Mait-MKI67 and CD8-Tef-GZMA were only mildly decreased (decrease was not significant) (Figure 3E). This may indicate that the decreased ratio of CD8-Tef-GZMA and the weaker tumoricidal capacity of CD8-Tef-APOC2 could influence the tumoricidal capacity of the CD8T subpopulations.

### 2.4. Pathway Analysis of CD8T Cells in Dominant Group vs. Nondominant Group

**Figure 4:**
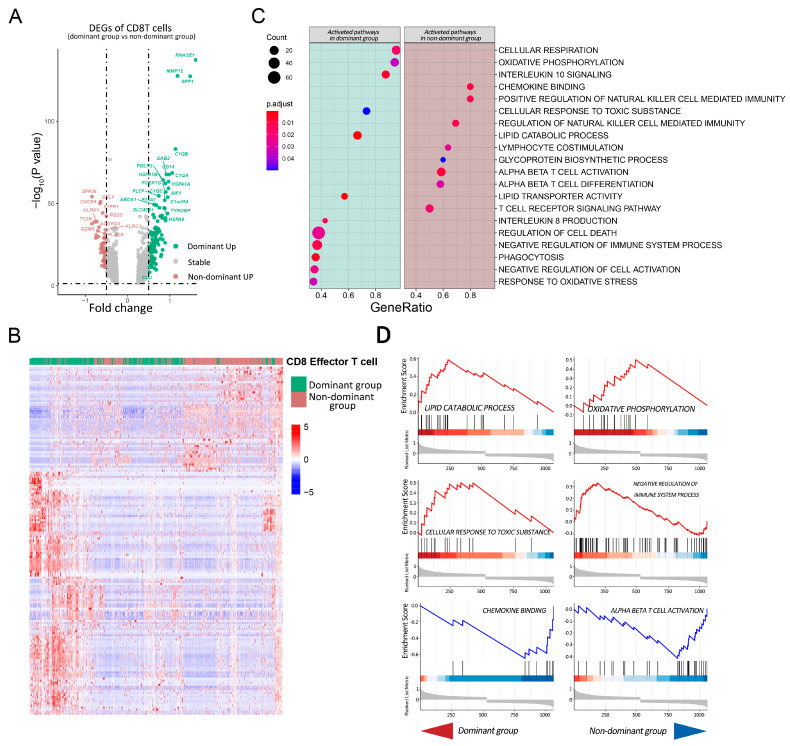
GSEA analysis of DEGs between CD8T cells in the dominant group and nondominant group. (**A**) Volcano plot of DEGs in CD8T cells of different populations. Green dots indicate genes upregulated in the dominant group and brown dots indicate genes upregulated in the nondominant group. The DEGs of CD8T cells in the TME of the dominant group and nondominant group are listed in Supplementary Table S6. (**B**) Heatmap of DEGs in CD8T cells of different populations. Rows indicate DEGs, columns are CD8T cells, and cell types are annotated above the heatmap. (**C**) Dot plot of gene set enrichment analysis (GSEA) showing enriched pathways of CD8T cells in the different groups. The size of each dot represents the enriched gene count in a pathway; the color of each dot indicates the significance test, with red showing greater significance. The results of the GO, REACTOME and HALLMARK for the dominant group and nondominant group are listed in Supplementary Table S7. (**D**) GSEA plots of 6 pathways are shown: 4 upper plots were activated in CD8T cells of the dominant group (enrichment score > 0), and 2 lower plots were activated in the nondominant group (enrichment score < 0).

A total of 1125 DEGs were extracted, among which 118 genes were upregulated in the dominant group, while only 51 genes were upregulated in the nondominant group (Figure 4A,B), and cell-killing-related genes, such as GZMK, GNLY and KLRK1, were specifically highly expressed, which was consistent with the above results. We used the gene set enrichment analysis (GSEA) approach for the 1125 DEGs to accurately quantify the pathway enrichment scores in the CD8 Tef cells (Figure 4C,D). In the nondominant group, the activation pathways of T cells and NK cells were upregulated, such as alpha–beta T cell activation, lymphocyte costimulation, chemokine binding, and the positive regulation of natural-killer-cell-mediated immunity; however, some immunosuppressive pathways were selectively activated in the dominant group, such as negative regulation of the immune system process, interleukin-10 signaling, and interleukin-8 production. At the same time, responses to oxidative stress and cellular responses to toxic substances were activated in the dominant group, which implies that CD8T cells in the dominant group faced stronger metabolic and survival pressures. For the metabolism of CD8T cells, activations of the lipid catabolic process and oxidative phosphorylation were observed in the dominant group, while no significant change in metabolic-related pathways was found in the nondominant group. In conclusion, the immune status of CD8T cells in the dominant group was suppressed, which was consistent with the results of the previous analysis.

### 2.5. CD8 Effector T Cells Have Different Metabolic Programs and Various Immune Statuses under the Influence of Glutamine Metabolism in Cancer Cells

**Figure 5:**
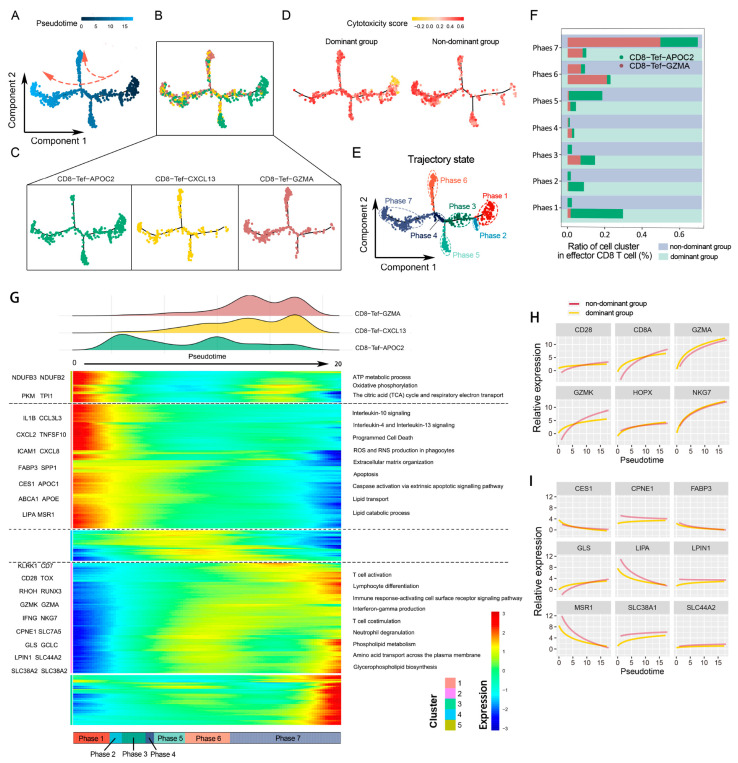
Differentiation directions of Tef subsets under different glutamine metabolic stress. (**A**) Pseudotime-ordered analysis of Tef cells. Arrows indicate direction of development. (**B**,**C**) Trajectory trees showing the distribution of Tef cell subtypes along the developmental trajectories. T cell subtypes are labeled by color: CD8-Tef-GZMA is colored with brown, CD8-Tef-CXCL13 with yellow, and CD8-Tef-APOC2 with green. (**D**) 2D pseudotime plot showing the dynamics of cytotoxic score of Tef cells in the dominant group and nondominant group. (**E**) 2D graph of pseudotime-ordered Tef cells. The trajectory state is labeled by color. (**F**) Histogram showing the cell distributions of CD8-Tef-GZMA and CD8-Tef-APOC2 over 7 phases between the dominant group and nondominant group. Tef subtypes are labeled by colors. (**G**) Heatmap showing the dynamic changes in gene expression along pseudotime. Representative genes of clusters 1, 2, and 5 are listed to the left of the heatmap. The DEGs of this heatmap are listed in Supplementary Table S8. Immune-regulation-related and metabolism-related pathways are marked to the right of the heatmap. Enriched pathways (GO and REACTOME) of the three clusters are listed in Supplementary Table S9. (**H**,**I**) Two-dimensional plots showing the dynamic expressions of metabolism-associated genes (**I**) and immune-associated genes (**H**) during Tef cell transitions along pseudotime.

With the above results, we found that the heterogeneity of CD8T cells in the two populations was mainly focused on two subsets: CD8-Tef-GZMA and CD8-Tef-APOC2. To further understand the gene and pathway dynamic changes in these subsets, we observed dynamic change in immunological status and the process of metabolism reprogramming by inferring the state trajectories with the Monocle package. The cells were sequenced using pseudotime 0 to 20 in a trajectory tree, and the trajectories with the lowest pseudotime were defined as the initiation phase (phases one to four), while the trajectories with the highest pseudotime were defined as the end phase (phases five, six and seven) (Figure 5A). We also visually displayed the distribution of the three CD8 Tef subsets in a cell trajectory tree (Figure 5B,C).

The analysis showed that CD8 Tef cells in the initiation phase with low cytotoxicity scores were mainly derived from patients in the dominant group, while CD8 Tef cells in the end phase with high cytotoxicity scores were mainly derived from the nondominant group (Figure 5D,E). We calculated the cell composition ratios of the two CD8 Tef subpopulations over seven phases. In the initiation phase, most of the CD8 Tef cells were mapped to CD8-Tef-APOC2 in the dominant group, while only a small part belonged to CD8-Tef-GZMA in the dominant group. During the pseudotime increase (phases 1 to 7), the CD8-Tef-APOC2 ratio in the dominant group decreased, and the ratio of CD8-Tef-GZMA in the nondominant group was gradually elevated (Figure 5F).

CD8-Tef-APOC2 was characterized by some activated pathways, such as oxidative phosphorylation and the positive regulation of lipid transport, and immunosuppressive pathways such as interleukin-10, interleukin-4, and interleukin-13 signaling were also significantly upregulated (Figure 5G). This implies that CD8-Tef-APOC2 was an immunosuppressed subpopulation that aggregated in the dominant group. This subpopulation showed lipid hypermetabolism, and the lipid transport-related genes of MSR1, FABP3, CES1, and LIPA were upregulated (Figure 5I). Therefore, we speculated that CD8-Tef-APOC2 tends to transport exogenous lipids to supplement the “energy gap”. During the pseudotime increase, the gene expressions of the lipid-transport-related pathway in the initiation phase (mostly CD8-Tef-APOC) gradually decreased, but the gene expressions, such as GLS, SLC44A2 and SLC38A1, relating to glycerophospholipid biosynthesis, phospholipid metabolism and amino acid transport across the plasma membrane in the end phase (mostly CD8-Tef-GZMA) were raised (Figure 5G,I). Simultaneously with metabolism reprogramming, the immune activation signaling pathways of CD8-Tef-GZMA were promoted, including lymphocyte differentiation, alpha–beta T cell activation, interferon gamma production, and the interleukin-2 family signaling pathway (Figure 5G). The genes upregulated in these pathways included CD28, CD8, GZMA, GZMK, NKG7, and HPOX (Figure 5H). These changes suggested that CD8-Tef-GZMA might prefer to take up a variety of amino acids, including glutamine, to promote the endogenous synthesis of glycerophospholipids and that this unique metabolic program facilitates proliferation and immune activation.

Overall, we tentatively realized that the metabolism reprogramming of CD8 Tef subpopulations may be strongly associated with the glutamine nutrition partitioning between cancer cells and CD8T cells and might be an important factor leading to the reshaping of the immunophenotype in CD8T cells. Therefore, we speculated that interfering with glutamine metabolism in the TME may contribute to the enhanced proliferation capacity of CD8T cell subpopulations and may increase the efficacy of immune checkpoint inhibitors.

### 2.6. Glutamine Metabolism Inhibitor (JHU083) Enhances the Efficacy of Immune Checkpoint Inhibitors (PD-1 Inhibitor)

**Figure 6:**
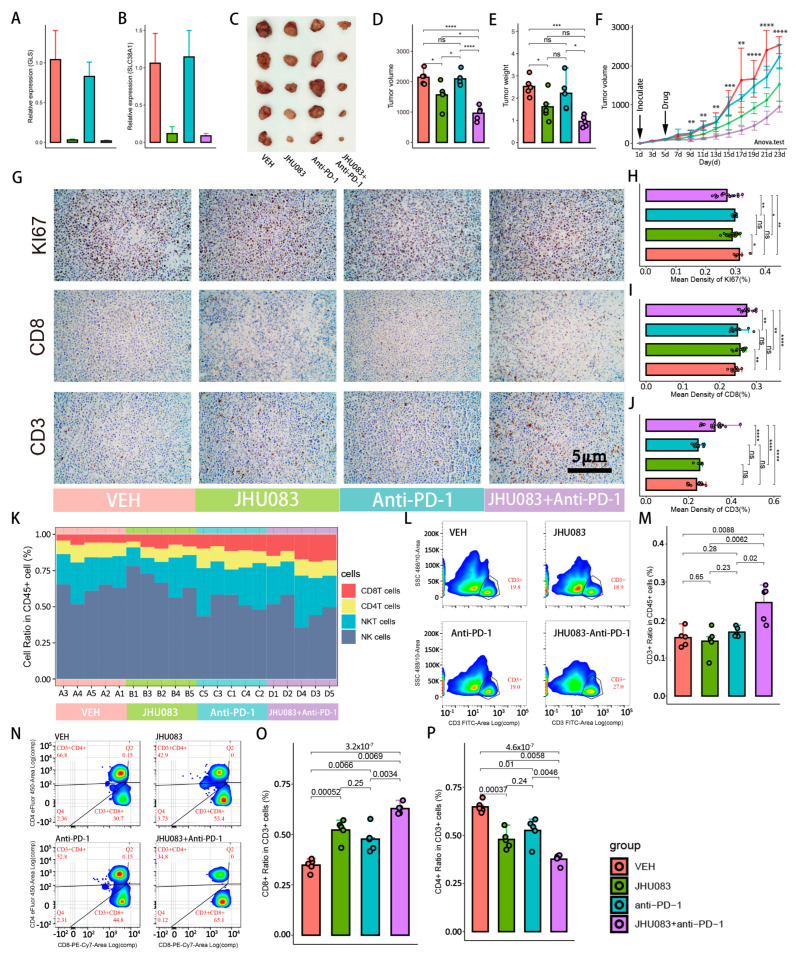
Glutamine metabolism inhibitors promote the proliferation of CD8+ T cells and improve response to immunotherapy. Groups are labeled with different colors: red, green, blue, and purple indicate VEH, JHU083, antiPD-1, and JHU083 + antiPD-1 groups, respectively. ns: no significance; * *p* < 0.05; ** *p* < 0.01; *** *p* < 0.001; **** *p* < 0.0001. (**A**,**B**) RNA relative expressions of GLS. The sequence of primer is listed in Supplementary Table S12. (**A**) and SLC38A1 (**B**) in tumor tissue after treatment with drugs. (**C**) Mouse subcutaneous tumors after inoculation with H22 cell for 22 days. (**D**,**E**) Box plots showing volumes (**D**) and weights (**E**) of tumor tissues of 4 groups after harvesting. The raw data on tumor volumes and weights are given in Supplementary Table S10. (**F**) Tumor growth curve illustrating the tumor growth rates of different groups. Inoculation with H22 cells occurred on the first day, and drug treatment occurred on the third day. Tumor volumes were measured every 2 days. Significance test was performed with ANOVA. Raw data on tumor volumes are given in Supplementary Table S11. (**G**) Immunofluorescence of Ki67, CD8, and CD3. Brown was considered to be strongly positive staining. (**H**–**J**) Box plots indicating immunohistochemical quantification of Ki67, CD8 and CD3. (**K**) Stacked bar plot showing dynamic changes in 4 immune cells sorted by the proportion of CD8+ T cells from small to large. Mouse numbers are marked on the *x*-axis. (**L**,**N**) Cell-sorting diagrams of flow cytometry, including T cells (**L**), CD8+ T cells, and CD4+ T cells (**N**). Antibodies used in flow cytometry are given in Supplementary Table S13. (**M**,**O**,**P**) Histograms showing changes in above cell ratios of 4 groups.

JHU083 is a precursor drug of the glutamine-metabolizing enzyme inhibitor DON, which inhibits a variety of intracellular enzymes related to the glutamine metabolic pathway, including glutaminase, glutamate dehydrogenase, NAD synthase, and asparagine synthase. Here, we compared the efficacy of four groups, including the JHU083 group, the PD-1 blocker group, the combined JHU083 and PD-1 blocker group, and the VEH control group. The mRNA expression levels of SLC7A5 and GLS were significantly decreased after treatment with JHU083, which demonstrated the successful inhibition of glutamine metabolism in subcutaneous hepatocellular carcinoma tumor tissue (Figure 6A,B). Compared with the VEH group, the volumes and weights of tumors in the JHU083 group were significantly decreased, while the efficacy of the PD-1 blocker group was not satisfactory (Figure 6C,F). However, distinct from the poor results of PD-1 blockers alone, JHU083 combined with PD-1 blockers could not only significantly inhibit tumor growth, but also showed a stronger efficacy than the JHU083 group. Next, we performed immunohistochemical staining of tumor tissues, including KI67, CD3, and CD8 (Figure 6G). There was no significant difference in KI67 between the VEH control and PD-1 blocker groups, suggesting that blockading of the PD-1 signal pathway alone could not inhibit the proliferation of tumor cells. However, the KI67 protein expression level of tumor cells was significantly decreased, both in the combined group of PD-1 blocker and JHU083 and in the JHU083 group (Figure 6H). With the exception of the JHU083 group and the PD-1 blocker group, only the combined group of JHU083 and PD-1 blocker was found to have a significant increase in the expression level of CD3 protein. For CD8 protein, we observed significant increases both in the combined group of JHU083 and PD-1 blocker and the JHU083 group, while a mild but not statistically significant increase was also found in the PD-1 blocker group (Figure 6I,J).

Because quantitative immunohistochemical assays are subject to subjectivity and cannot precisely measure cell numbers, we performed flow cytometry to accurately count the abundance of CD8T cells in the tumor tissue. We found a significant increase in T cells for the combined group of PD-1 blocker and JHU083 compared with the individual JHU083 and PD-1 blocker groups (Figure 6L,M). We also counted the numbers of CD8T cells and CD4T cells in the different groups (Figure 6N–P). We detected a higher CD8T cell proportion and a lower CD4T cell proportion in the combined PD-1 blocker and JHU083 group compared with the individual PD-1 blocker and JHU083 groups, but the numbers of CD8T cells and CD4T cells between the PD-1 blocker group and the JHU083 group were not statistically different. Other immune cells, including NKT cells and NK cells, did not change significantly after the administration of the drugs. Sequential changes in the CD8T cells, CD4T cells, NK cells, and NKT cells between the four groups are shown in Figure 6K.

Taken together, these results suggest that after the application of the glutamine metabolism inhibitor JHU083, PD-1 blockers demonstrated dramatic efficacy for tumor growth inhibition. In the immune microenvironment where glutamine metabolism was blocked, the growth of cancer cells was inhibited, while T cells, especially CD8T cells, were more proliferative.

## 3. Discussion

Hepatocellular carcinoma is a malignant tumor with a five-year survival rate of less than 10% [9,10]. Although immune checkpoint inhibitors such as pabolizumab and atilizumab have improved the overall survival rate of hepatocellular carcinoma patients, their response rate is still unsatisfactory [11]. Therefore, the intensive investigation of the formation mechanism of a tumor-infiltrating immunosuppressive microenvironment is an important approach for improving therapy for hepatocellular carcinoma. In this study, we found that the higher the glutamine-metabolism-related gene expression scores of cancer cells, the worse the prognoses of patients. Meanwhile, a single-cell sequencing analysis showed that immunosuppressive microenvironments of HCC occurred in patients whose glutamine-metabolism-related gene expression scores in cancer cells were significantly stronger than those of CD8T cells, although not in relation to the absolute strength of glutamine-metabolism-related gene expression scores of cancer cells. Moreover, the inhibition of glutamine metabolism in the hepatocellular carcinoma microenvironment improved antitumor immune responses and enhanced the efficacy of immune checkpoint inhibitors.

Unbalanced nutrient partitioning between tumor cells and immune subpopulations is an important driver of immune heterogeneity among cell subpopulations. For example, the strong fat metabolism of MC38 cells suppressed the fat uptake capacity of T lymphocytes and impaired immune function [12], and the uptake and consumption of arginine by tumor cells have been found to significantly inhibit the antitumor capacity of lymphocytes [13,14,15]. In the single-cell dataset, we found that the glutamine metabolism gene expression scores of cancer cells did not significantly correlate with the antitumor capacity of CD8T cells. However, further analysis revealed that glutamine metabolism gene expression scores were significantly higher in cancer cells than in CD8T cells in a portion of the patients. Therefore, according to the differences in the glutamine metabolism gene expression scores between tumor cells and CD8T cells in each patient, we divided these patients into cancer-cell-dominant and nondominant groups. Further analysis revealed a significant decrease in antitumor factors (GZMA, GZMB, NKG7, and PRF1) in CD8T cells and an increase in the proportion of cancer cells, especially in the cancer-cell-dominant group. To analyze the reasons for the decreased tumoricidal capacity in CD8T cells, we analyzed the CD8T subpopulation. We observed a significant decrease in the proportion of the CD8-Tef-GZMA subpopulation and a significant downregulation of cytotoxicity scores in the CD8-Tef-APOC2 subpopulation within the dominant group, while other subpopulations did not differ. Therefore, the immunosuppressive tumor microenvironments in patients of the dominant group could be caused by the CD8-Tef-GZMA and CD8-Tef-APOC2 subpopulations.

A genetic dynamics pseudotime analysis and a GSEA revealed that immune remodeling of tumor-infiltrating CD8T cells was accompanied by the reprogramming of metabolic patterns. During the remodeling of the immunosuppressive CD8-Tef-APOC2 subpopulation into a tumor-killing CD8-Tef-GZMA subpopulation, the metabolic pattern of exogenous lipid metabolism was gradually replaced by the metabolic patterns of amino acid and endogenous lipid synthesis. The CD8-Tef-APOC2 subpopulation, which was heavily infiltrated in the TME of the cancer-cell-dominant group, was characterized by the upregulation of the exogenous lipid metabolism and immunosuppressive pathways. The proportion of the CD8-Tef-GZMA subpopulation was significantly elevated in the cancer cells nondominant group, and this process was accompanied by the upregulation of amino acid metabolism and endogenous lipid synthesis [16]. Existing research proved that tumor cells consume most of the nutrients in the immune microenvironment, while CD8T cells only consume less than 5% of glutamine [4]. At the same time, the enrichment of the exogenous lipid metabolism pathway in patients in the dominant group suggested that CD8T cells are likely to ingest exogenous fatty acids as alternative energy to maintain metabolism. This unique lipid metabolism mechanism has been confirmed in many tumors [17,18,19] and may be related to the fact that lipolysis-derived ketobodies can provide energy faster than other metabolic substrates in an anaerobic environment [20].

Blockading the PD-L1/PD-1 signaling pathway has been demonstrated as an important means to break tumor immune escape in a large number of clinical drugs [21,22,23], but sufficient immune cells are also a prerequisite for the sustained action of PD-1 blockers [24,25,26]. The results of the single-cell RNA-seq data suggest that interfering with the glutamine metabolism of tumor cells and CD8T cells may produce a positive effect on the tumor-killing ability of CD8T cell subsets. In our subcutaneous tumor model of mice, we demonstrated that a glutamine metabolism inhibitor increased the number of CD8T cells in the immune microenvironment and enhanced the inhibitory effect on tumor growth of a PD-1 blocker. In the glutamine metabolism inhibitor group, the volumes and weights of subcutaneous tumors were significantly decreased. Furthermore, the immunohistochemical results also suggested that tumor cell proliferation was inhibited and was accompanied by the significant proliferation of CD8T cells, and the flow cytology results were consistent with the immunohistochemical results. In the PD-1 inhibitor group, we did not observe either the growth inhibition of subcutaneous tumors or the significant proliferation of CD8T cells. However, we found that PD-1 inhibitors in the combination group exerted stronger growth inhibition of tumor cells and greater proliferation of CD8T cells than PD-1 inhibitors alone, and this effect was even stronger than that of JHU083. Combined with the results of immunohistochemistry and flow cytometry, we speculated that the excellent effects may be closely related to the growth inhibition of tumor cells and the proliferative effect on CD8T cells of JHU083. The distinct effects of JHU083 on these two cell subpopulations have been found and confirmed in colon cancer research. Under the overall inhibition of the glutamine metabolic pathway, CD8T cells can still uptake compensatory glucose, increase PC enzymatic activity, and enhance the activity of the acetate metabolism pathway to guide carbon sources into the TCA cycle and increase intracellular metabolism [27]. In addition, the inhibition of GLS can reduce the accumulation of α-ketoglutarate, which has been proved to promote CD8T cell differentiation to memory cells, giving CD8T cells the phenotypic characteristics of high proliferation and long life [28,29]. However, this flexible metabolic compensation mechanism was also shown to be lacking in tumor cells [27].

Additionally, a shortcoming of this study is that, although we demonstrated that the inhibition of glutamine metabolism in the TME induced the proliferation of CD8T cells, we could not exclude whether JHU083 regulated CD8T cells by affecting other immune cell subpopulations, such as CD4Trg or MDSCs. The specific regulatory mechanism of glutamine nutrient partitioning between CD8T cells and tumor cells for reshaping tumor immunity also needs further experimental validation. Finally, the animal model we used only partially summarized the pathological and clinical characteristics of human tumors, so the sensitizing effect of glutamine metabolism inhibitors on PD-1 blockers also needs further validation in human clinical trials.

In summary, we proposed a new tumor metabolic microenvironment signature at the transcriptome level that could affect the immune function of CD8T cells, namely the glutamine nutrient partitioning between cancer cells and CD8T cells. Specifically, when the glutamine metabolism gene expression scores of tumor cells exceeded those of CD8T cells at the transcriptome level, we found that immunosuppressive CD8T cell subsets were enriched. This suggested that interference with the glutamine metabolism of the immune microenvironment could be beneficial to enhance the efficacy of immune checkpoint inhibitors. Our animal model of liver cancer verified our conjecture. JHU083 in combination with a PD-1 inhibitor exhibited excellent tumor-suppressive effects. We noticed that the number of tumor-infiltrating CD8T cells increased after treatment with glutamine metabolic inhibitors, which could be the reason for the increased efficacy of the PD-1 inhibitors, but the potential regulatory mechanism needs further study. Here, we proposed a new target to improve the number of tumor-infiltrating CD8T cells in HCC patients and provided new ideas for further research on metabolism combined with immunotherapy.

## 4. Materials and Methods

### 4.1. Data Resources for scRNA-Seq and Bluk RNA-Seq

CNP0000650 single-cell RNA sequencing data (https://db.cngb.org/search/project/CNP0000650/ (accessed on 15 March 2022)) [30] were downloaded from the China National GeneBank (CNGB), and 10 patients with primary hepatocellular carcinoma were selected (2 patients with fewer than 10 tumor cells were excluded). First, cells with gene counts greater than 50 and genes expressed in three or more cells were included. Then genes with mitochondrial gene proportions ≥ 4%, ribosomal gene proportions ≤ 2%, and hemoglobin gene proportions ≥ 10% were excluded. The remaining eligible cells and genes were filtered for downstream analysis (Figure S1).

FPKM data of bulk RNA-seq data and clinical follow-up data from TCGA-LIHC (https://portal.gdc.cancer.gov/ (accessed on 22 February 2022)) were downloaded for clinical analysis. After excluding patients without survival information and expression matrices, 363 transcriptional profiles of tumor tissue, 11 clinical pathological parameters corresponding to patients (T-stage, age, degree of inflammation of paracancerous tissue, plasma AFP value, cirrhosis, tumor grade, vascular invasion, tumor margin, history of hepatitis, alcohol consumption, and smoking), and 2 survival indicators (survival status and survival time) were included (Table S1). All patients were diagnosed with hepatocellular carcinoma after pathological examination.

### 4.2. Data Processing and Analysis of TCGA Cohort

The ‘ssGSEA‘ function was used to assess the cancer cell glutamine-metabolism-related gene expression scores of each patient for glutamine-metabolism-related genes (ALDH18A1, GCLC, GCLM, GLS, GLUD1, GOT2, MTHFS, SLC38A1 and SLC38A2). Similarly, due to the specific expression of cytotoxicity-related genes in immune cells, the bulk transcriptome data could also be used to calculate the cytotoxicity score of the immune population in each patient to assess their antitumor activity. The median value of the glutamine-metabolism-related gene expression scores was defined as the cut-off value, and the 363 patients were divided into a high score group and a low score group to explore the relationship between cancer cell glutamine-metabolism-related gene expression scores and prognosis. To investigate the effects of glutamine-metabolism-related gene expression scores and other clinical factors on prognosis in HCC patients, we performed univariate (screening criteria: combined with clinical background and *p* < 0.2) and multivariate (screening criteria: *p* ≤ 0.05) Cox proportional hazards regression (CPHR) analyses in the TCGA cohort.

### 4.3. Data Processing and Subgroup Annotation of Single-Cell RNA-Seq

The Seurat package in R (version 3.0, Ross Ihaka, Auckland, New Zealand) was used for single-cell RNA-seq data. By applying the ‘NormalizeData’ function of the Seurat package, the expression matrix of all the cells was normalized with a scale factor of 10,000, and 3000 highly variable genes were filtered with the ‘FindVariableFeatures’ function using the ‘vst’ method. Next, the ‘ScalData’ function scaled the data, and all genes were set as reference genes. PCA analysis was used to identify significantly available dimensions with *p*-value < 0.05. We operated a t-distributed stochastic neighbor embedding (t-SNE) algorithm to reduce the dimensionality of 15 PCs and then performed cluster classification analysis across all the cells. The clustered cells were annotated as different biologic subpopulations according to known molecular markers summarized in the previous literature, including T cells (CD3E and CD3D), B cells (CD19), NK cells (NCAM1, KLRD1 and FCGR3A), myeloid cells (CD163 and CD86), and tumor cells (EPCAM, KRT18 and KRT8).

T cell subpopulations were extracted individually, and batch effects of T cells were corrected using the ‘Harmony’ package to ensure penalties for any specified unwanted technical or biological factors. Then, the same standardized and dimensional reductions were performed on the T cells. The ‘FindNeighbors’ and ‘FindClusters’ functions were performed with resolutions of 0.8–2 to identify individual cell clusters. The biological background of each cell cluster was defined by molecular markers supported by the available literature. The cited literature for each subpopulation and corresponding molecular markers is listed in Supplementary Table S2.

### 4.4. Calculating Pathway Enrichment Scores and Patient Grouping

Glutamine-metabolism-related gene expression scores of tumor cells ($GS_{tumor}$) and CD8T cells ($GS_{immune}$) were calculated with the ‘ssGSEA’ algorithm of the GSVA package based on the expressions of glutamine-metabolism-related genes (ALDH18A1, GCLC, GCLM, GLS, GLUD1, GOT2, MTHFS, SLC38A1, and SLC38A2). We performed two methods to group patients. First, we divided the patients into the glutamine-metabolism-related gene expression high score group (high score group) and the glutamine-metabolism-related gene expression low score group (low score group) according to the median value of $GS_{tumor}$ for each patient. Second, we calculated the score differences in glutamine-metabolism-related genes between cancer cells and CD8T cells as the dominance score for each patient. Patients whose glutamine-metabolism-related gene expression scores were significantly higher in tumor cells than in CD8T cells were defined as the cancer cell glutamine-metabolism-dominant group (dominant group), and patients with the opposite proportions were in the cancer cell glutamine-metabolism-nondominant group (nondominant group). After grouping, the cytotoxicity scores of CD8T cells between the two groups were also calculated and compared according to the expressions of cytotoxicity-related genes (GZMA, GZMB, PRF1, IFNG, EOMES, and NKG7).

$$\mathrm{Dominance}\;\mathrm{Score}=\;\mathrm{Median}\;\left(GS_{tumor}\right)-\;\mathrm{Median}\;\left(GS_{immune}\right)$$

### 4.5. Immunophenotype Remodeling and Metabolism Reprogramming of Tumor-Infiltrated CD8 T Cells in Dominant and Nondominant Groups

In order to clarify the difference in the tumoricidal capacity of CD8T cells, we further compared the expression levels of the cytotoxic genes of CD8T cells and compared the proportion changes in tumor cells for the 2 groups. Next, we investigated the changes in cytotoxicity scores and cell ratios in 4 CD8T subpopulations between the 2 groups. To further investigate the pathway changes of effector CD8T cells (Tef) undergoing glutamine metabolic stress in cancer cells, we applied the Monocle2 package in R to perform a genetic dynamics pseudotime analysis of CD8 effector CD8T cells. Monocle2 performed a reversed graph-embedding algorithm with a semiclustering machine learning method to simulate the cell development process and establish a “one root and multibranch” differentiation tree, where cells in the same branch shared the same characteristics of gene expression patterns and the same differentiation state. We extracted the gene expression matrix, gene information, and cell phenotype information of CD8 effector CD8T cells to construct CellDataSet objects. The ‘estimateSizeFactors’ function helped us to normalize the differences in the mRNA matrices. Upregulated different expression genes (DEGs) of effector CD8T cells displaying two metabolic patterns were screened (*p*-value < 0.05 and average logFC > 0.25) with the ‘FindAllMarkers’ function. Then, single cells were projected into the space and ordered into a trajectory with branch points using the ‘DDRTree’ method.

### 4.6. Gene Set Enrichment Analysis of CD8T Cells

A gene set enrichment analysis (GSEA) is a statistical method used to calculate distribution trends according to ranked genes correlating with phenotype and genes on predefined gene lists to determine their contributions to the phenotype. Compared with the classical GO and KEGG enrichment analyses, GSEA avoids the subjective bias of setting artificial thresholds to retain more valid information and quantifying the activity statuses of gene sets. We downloaded the gene sets of C2: CP: KEGG, C2: CP: REACTOME, and C5: GO (including BP, MF and CC) from MsigDB (https://www.gsea-msigdb.org/gsea/msigdb/ (accessed on 11 April 2022)). After extracting CD8T cell subpopulations, the ‘FindAllMarkers’ function was conducted to filter upregulated and downregulated DEGs in the dominant group, and their corresponding expression fold changes were calculated. We used the GSEA function of the clusterProfiler package to analyze the sequenced DEG list and obtain different pathways of CD8T cells in the 2 populations.

### 4.7. Animal Experiments

Thirty C57BL/6 mice (female: 15; male: 15) were purchased from Beijing Huafukang Biotechnology Co. and fed in a barrier system at the Animal Experimentation Center of Southwest Medical University. Mice of 8–12 weeks of age were used in all the experiments. The animal experiments were approved by and conducted under the supervision of the Animal Experimentation Ethics Committee of the Affiliated Hospital of Southwest Medical University.

To establish a subcutaneous tumor-forming animal model, 0.1 mL H22 cell line with a concentration of 1 × 10^6^/mL was inoculated into the right groins of mice. After five days of feeding, the long and short diameters of subcutaneous tumors were measured and tumor volume was calculated as V (mm3) = length (mm) ∗ width (mm) ∗ width (mm) ∗ π/6. Twenty mice with no statistical differences in subcutaneous tumor volume were selected and randomly divided into four groups: (I) the blank control group (VEH) which received a gavage of 100 μL 0.9% saline (once daily for 17 days) and an intraperitoneal injection of 100 μL 0.9% saline (once every 3 days for 17 days); (II) the glutamine metabolism inhibitor group (JHU083), which received a gavage of JHU083 dissolved in 100 μL saline (1 mg/kg/d once daily) and an intraperitoneal injection of 100 μL saline (once every 3 days for 17 days); (III) the immune checkpoint inhibitor group (PD-1 blocker group), which received an intraperitoneal injection of PD-1 blockers dissolved in 100 μL saline (1 mg/kg/d every 3 days) and a gavage of 100 μL 0.9% saline (once daily for 17 days); and (IV) the combined group of PD-1 blocker with JHU083, which received a gavage of JHU083 dissolved in 100 μL saline (1 mg/kg/d once daily) and an intraperitoneal injection of PD-1 blockers dissolved in 100 μL saline (1 mg/kg/d once every 3 days for 17 days).

Tumor volumes were measured every 2 days, and all mice were dosed for 17 days. Mice were euthanized on day 22, and subcutaneous tumor specimens were harvested for PCR, immunohistochemistry, and flow cytometry. The preparation and experimental procedure relating to the tumor tissues are described in the Supplementary Materials.

## Data Availability

Publicly available datasets were analyzed in this study. These data can be found at: TCGA-LIHC (https://portal.gdc.cancer.gov/ (accessed on 22 February 2022)) and CNP0000650 (https://db.cngb.org/search/project/CNP0000650/ (accessed on 15 March 2022)).

## Supplementary Materials

The following supporting information can be downloaded at: https://www.mdpi.com/article/10.3390/cells11233924/s1, supplementary experimental detailed methods; Figure S1: Quality assurance of single-cell sequencing data; Figure S2: Dimensionality reduction and clustering of single-cell sequencing data; Figure S3: t-SNE plots of marker genes in the main population; Figure S4: t-SNE plots of marker genes in the subpopulations; Table S1: Clinical parameters of patients in the TCGA-LIHC cohort; Table S2: molecular markers of main clusters and subpopulations; Table S3: Univariate and multivariate Cox proportional hazards regression analyses in the TCGA cohort; Table S4: Patient grouping; Table S5: Metadata of CD8+ T cells in the TME of 10 primary hepatocellular carcinoma patients; Table S6: DEGs of CD8 + T cells in the TME for dominant group and nondominant group; Table S7: The results of GSEA (GO, REACTOME and HALLMARK) for the dominant group and nondominant group; Table S8: DEGs in 5 clusters; Table S9: The enriched pathways (GO and REACTOME) of DEGs in 3 clusters; Table S10: Tumor volumes and weights in 4 groups after harvesting; Table S11: Raw data of tumor volumes; Table S12: primers used in study; Table S13: Antibodies used for flow cytometry. References [31,32,33,34] are cited in the supplementary materials.
