# Supplementary material for: Unbalanced Glutamine Partitioning between CD8T Cells and Cancer Cells Accompanied by Immune Cell Dysfunction in Hepatocellular Carcinoma
Source: Cells. 2022 Dec 4;11(23):3924. doi: 10.3390/cells11233924 (PMC9739589; doi:10.3390/cells11233924)
Supplement: Supplementary file 1 [file cells-11-03924-s001.zip › Supplementary experimental detailed methods.pdf]

Quantitative real-time polymerase chain reaction.

Approximately 50mg tumor tissue was grind and crushed, add an appropriate amount of Trizol lysis solution to it and lyse it thoroughly on ice. The lysate was then transferred to an enzyme-free EP tube and centrifuged at 4°C, 12,000 rpm/min for 10 min; the supernatant obtained by centrifugation was then transferred to another EP tube, chloroform was added, the supernatant and chloroform were mixed and left to stand for 15 min, next centrifuged at 4°C, 8,000 rpm/min for 15 min. Wash with 75% ethanol solution, centrifuge for 15 min at 4°C at 12000 rpm/min, add 20 μl DEPC water to the precipitate, wait for the precipitate to dissolve, measure the mRNA concentration. mRNA was collected and reverse transcribed into cDNA, which were amplified in triplicate using SYBR Green PCR Master Mix (Guangzhou RiboBio Co), 10 pmol of primer (Table S12), and 20 ng of cDNA per reaction with the StepOnePlus (Roche LightCycler 96). Quantitation was performed using the ΔΔCt method.

Immunohistochemistry (IHC).

All pathological diagnoses were made independently by 2 senior physicians in the Department of Pathology at the Southwest Medical University Hospital, and controversial diagnoses were assessed by a third physician and then decided by joint consultation. The specific steps of staining were as follows.

(1) Dewaxing and hydration: The slices were placed in the oven at a temperature of 60°C for 90 min, then placed in xylene for 30 min for dewaxing, then the slices were immersed in ethanol (anhydrous ethanol, 95% ethanol, 75% ethanol) in a gradient from high to low concentration for 5 min, and finally rinsed repeatedly with double-distilled water for 5 min.

(2) Antigen repair and peroxidase removal: The treated tissue sections were placed in a repair cassette with 200 ml of ethylene glycol tetraacetic acid (EDTA) solution, then placed in an autoclave with double-distilled water, first heated to vapour, then allowed to cool, and then rinsed with double-distilled water. The sections were then placed in 3% hydrogen peroxide solution (H2O2) for 10 min incubation protected from light, allowed to cool and then soaked 3 times with double distilled water for 5 min each and rinsed with PBS for 5 min.

(3) Addition of antibody, colour development, re-staining and blocking: sections were added dropwise with antibody (KI67, CD3 and CD8) diluted at 1:200 and refrigerated overnight at 4°C.

The next day the sections were washed three times with PBS for 5 min each time. Second day, the sections were washed three times with PBS for 5 min each time, shaken dry, incubated with secondary antibody for 30 min, and washed three times with PBS for 5 min each time. The reaction was terminated by adding a drop of DAB staining solution to the sections and observing a positive reaction under the microscope. After washing, the sections were fractionated with ethanol hydrochloride solution, then washed, dehydrated, sealed and labelled.

(4) After the above steps were completed, the pathological sections were observed under an inverted fluorescent microscope. The expression levels of KI67, CD3 and CD8 proteins were measured with Image J software.

Flow cytometry.

Immune cell populations were identified via flow cytometry from respective dissociated whole tumor cell suspensions.

(1) After mechanically cutting the tumor tissue, it was filtered with 300 mesh filter cloth, centrifuged with 300g for 5min, and the cell concentration was adjusted to 10*6 /ml with PBS. (2) 1 μg antibody (CD8a, CD3, cd49b, CD45, CD4, LIVE/DEAD) (Table S13) were add into 100 μL cell suspension in the sterile EP tube. Dye at 4 ℃ for 30 min without light after mixing. (3) Adding 1000 μL PBS to wash the mixture, the supernatant was removed after centrifuging with 300g for 5min. (4) Cells were resuspended by 400 μL PBS and then detected by ZE5 flow cytometry, flow cytometry data were analyzed using FlowJo software.
