# Supplementary figures and images for: Unbalanced Glutamine Partitioning between CD8T Cells and Cancer Cells Accompanied by Immune Cell Dysfunction in Hepatocellular Carcinoma
Source: Cells. 2022 Dec 4;11(23):3924. doi: 10.3390/cells11233924 (PMC9739589; doi:10.3390/cells11233924)

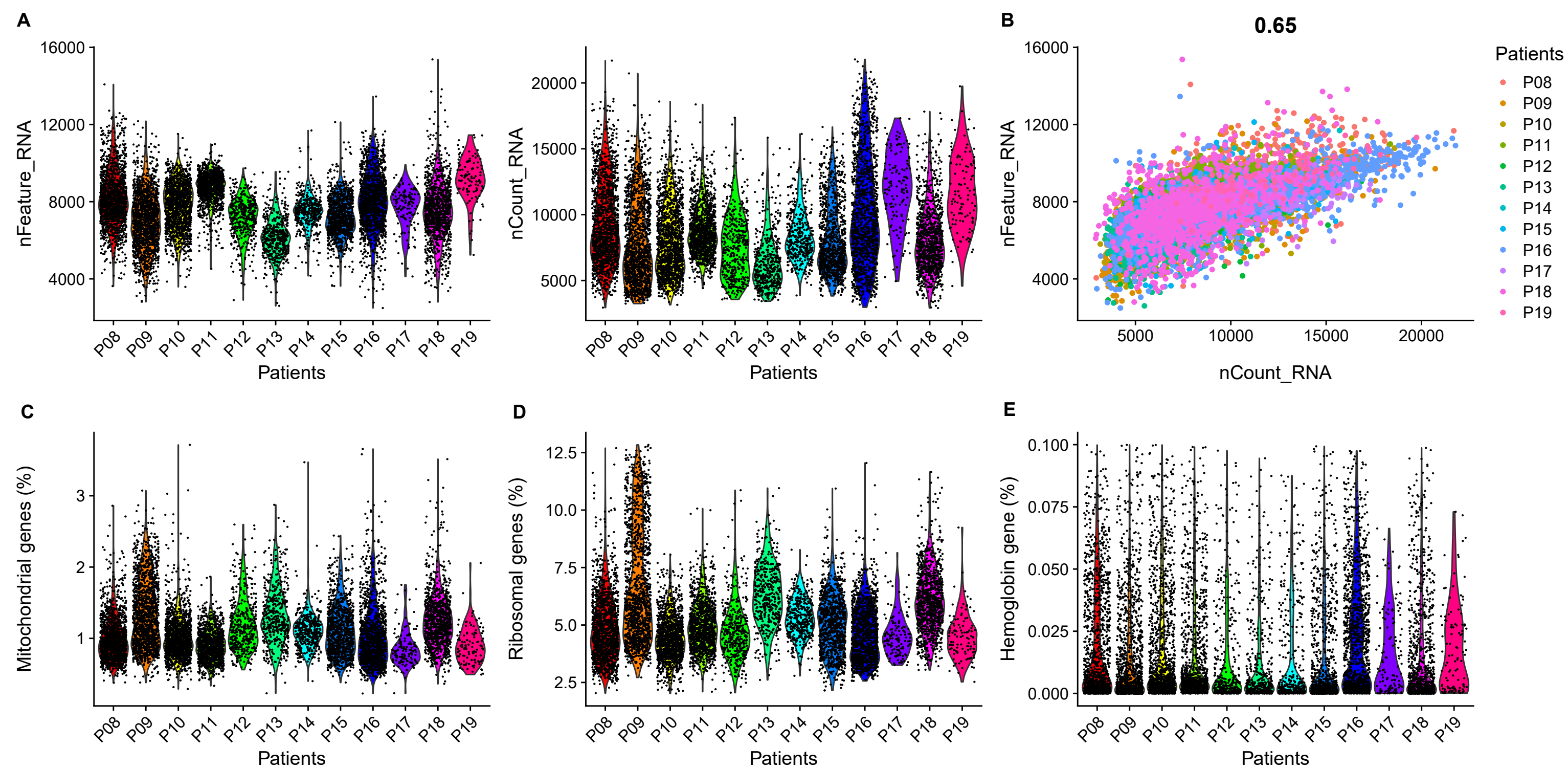

Supplement: Supplementary file 1 [file cells-11-03924-s001.zip › Figure S1 Quality Control.pdf]

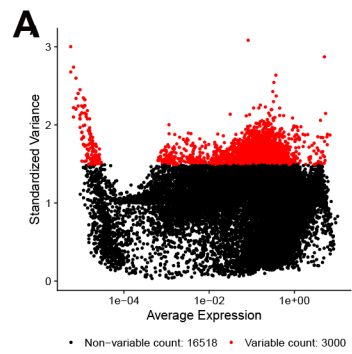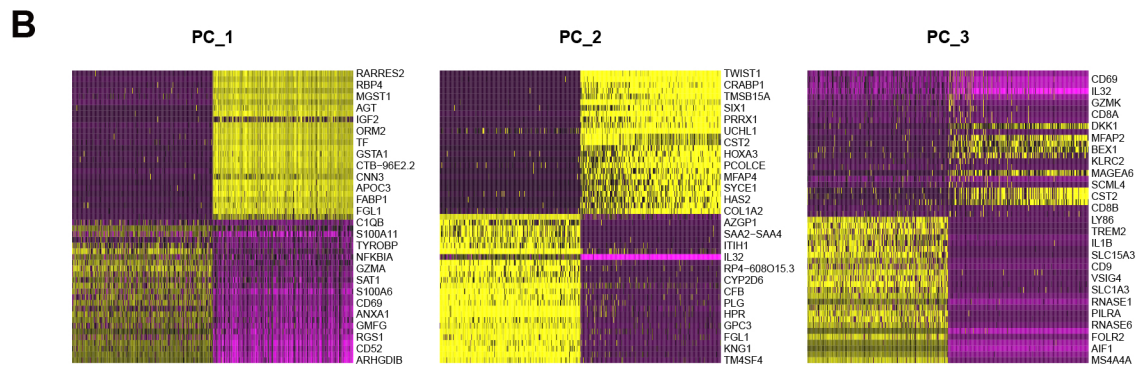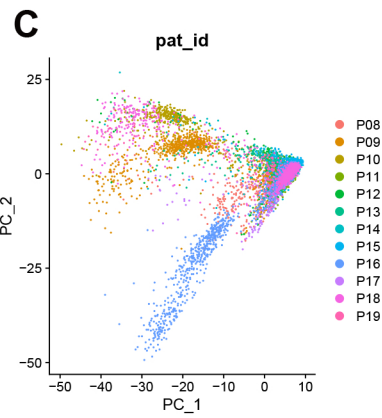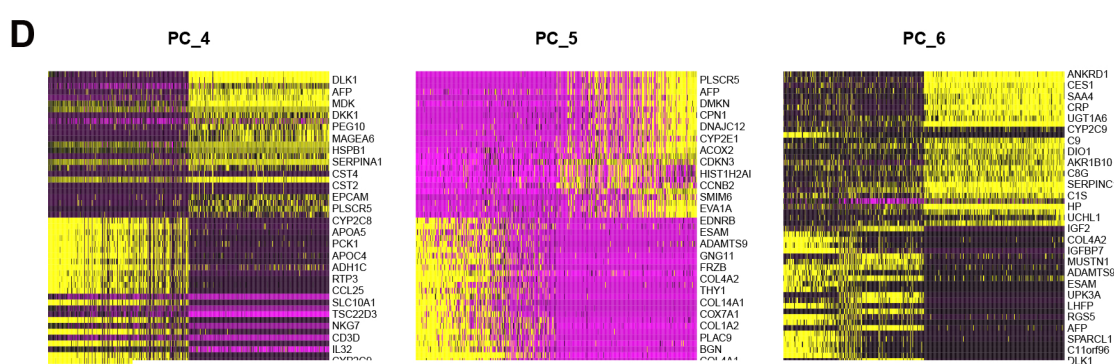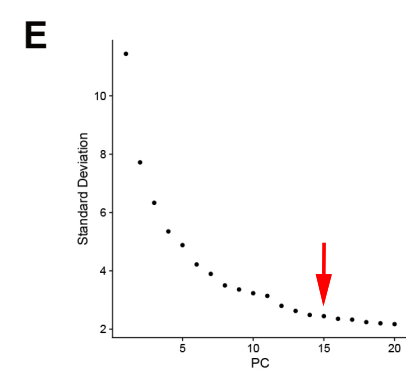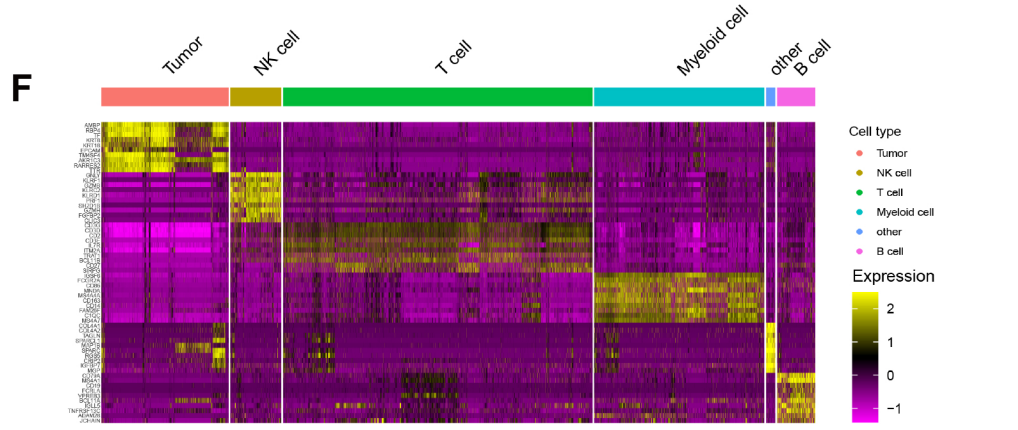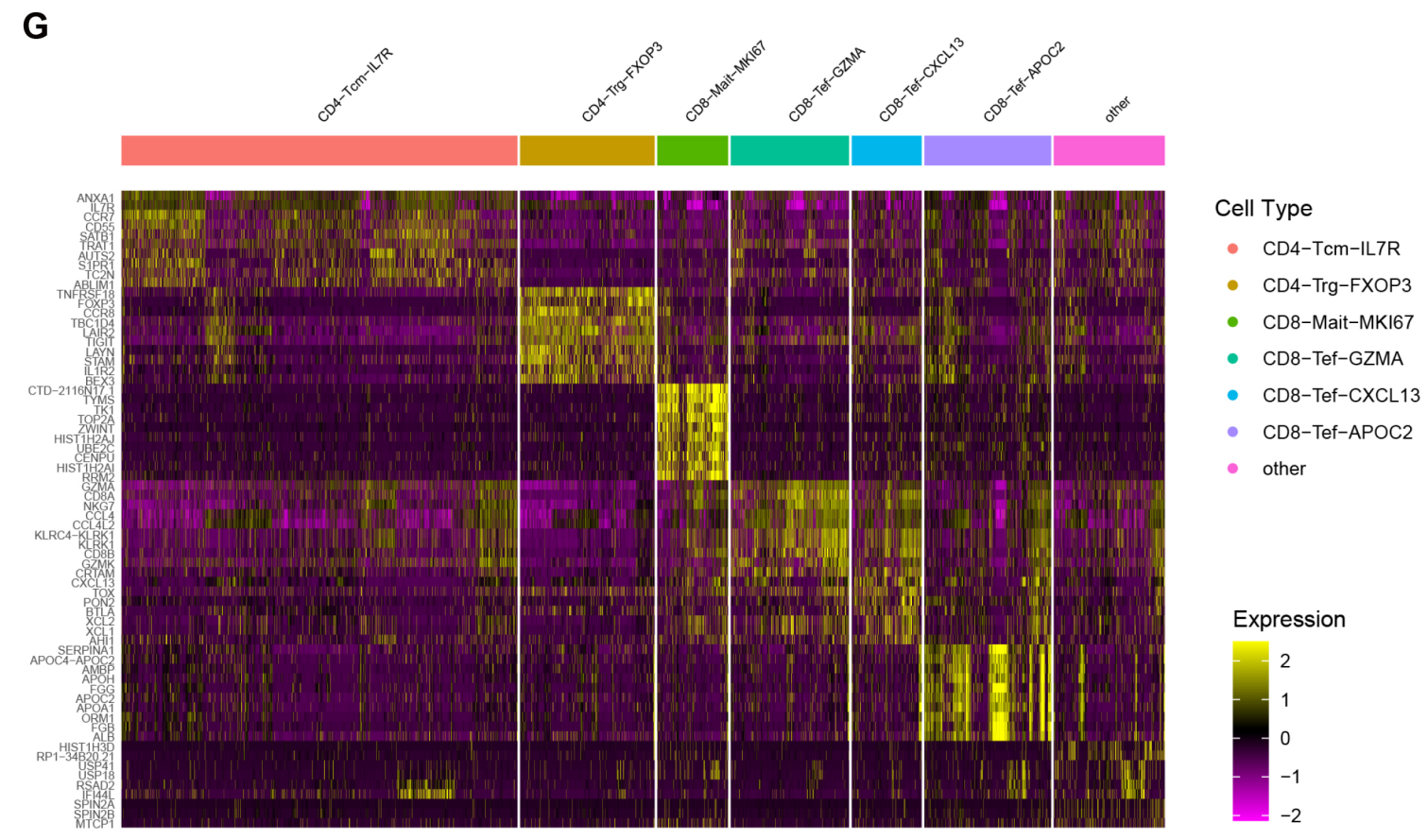

Supplement: Supplementary file 1 [file cells-11-03924-s001.zip › Figure S2 Single Cell Sequencing Supplementary File.pdf]

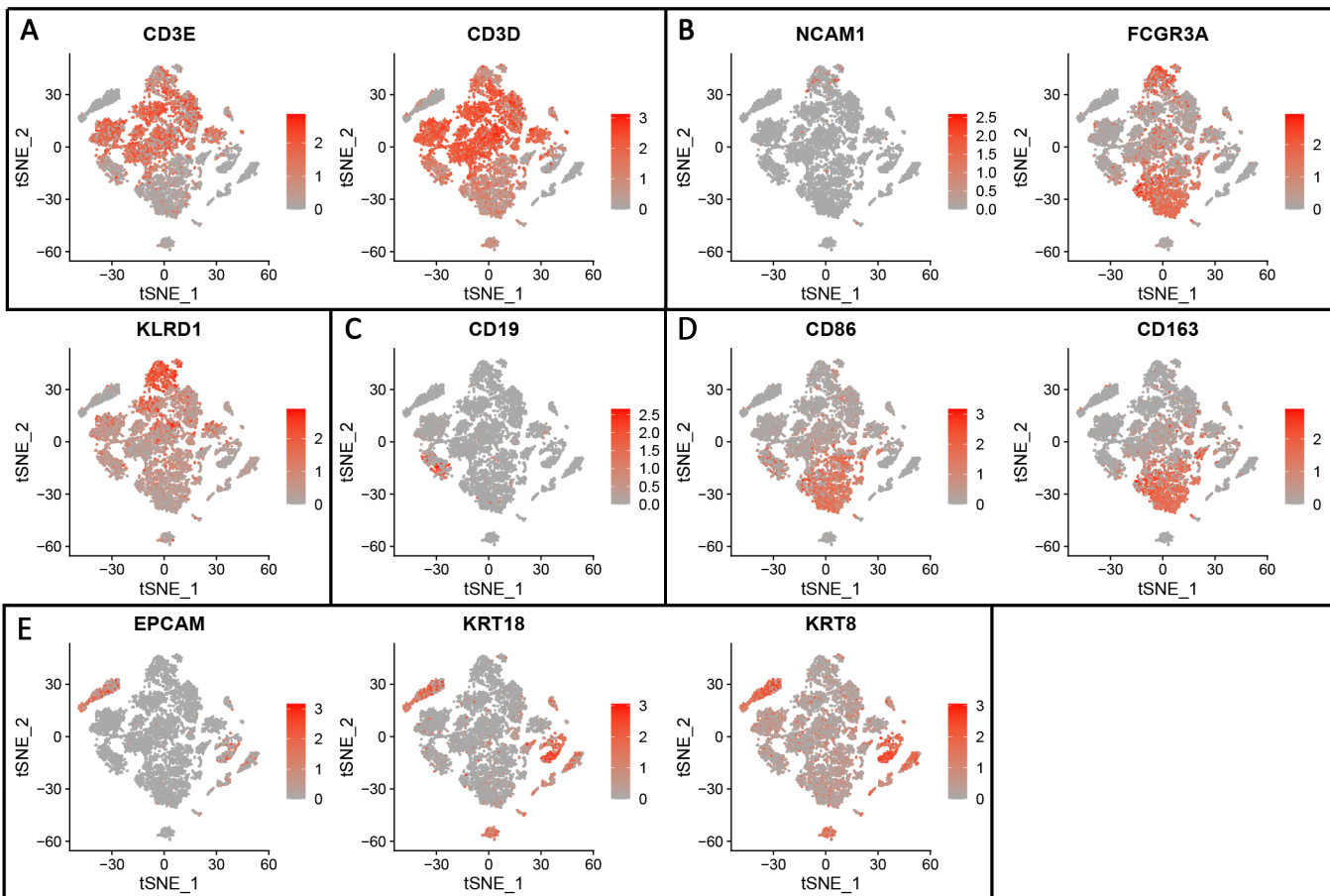

Supplement: Supplementary file 1 [file cells-11-03924-s001.zip › Figure S3 bidcluster_supply_merkergene_dimplot.pdf]

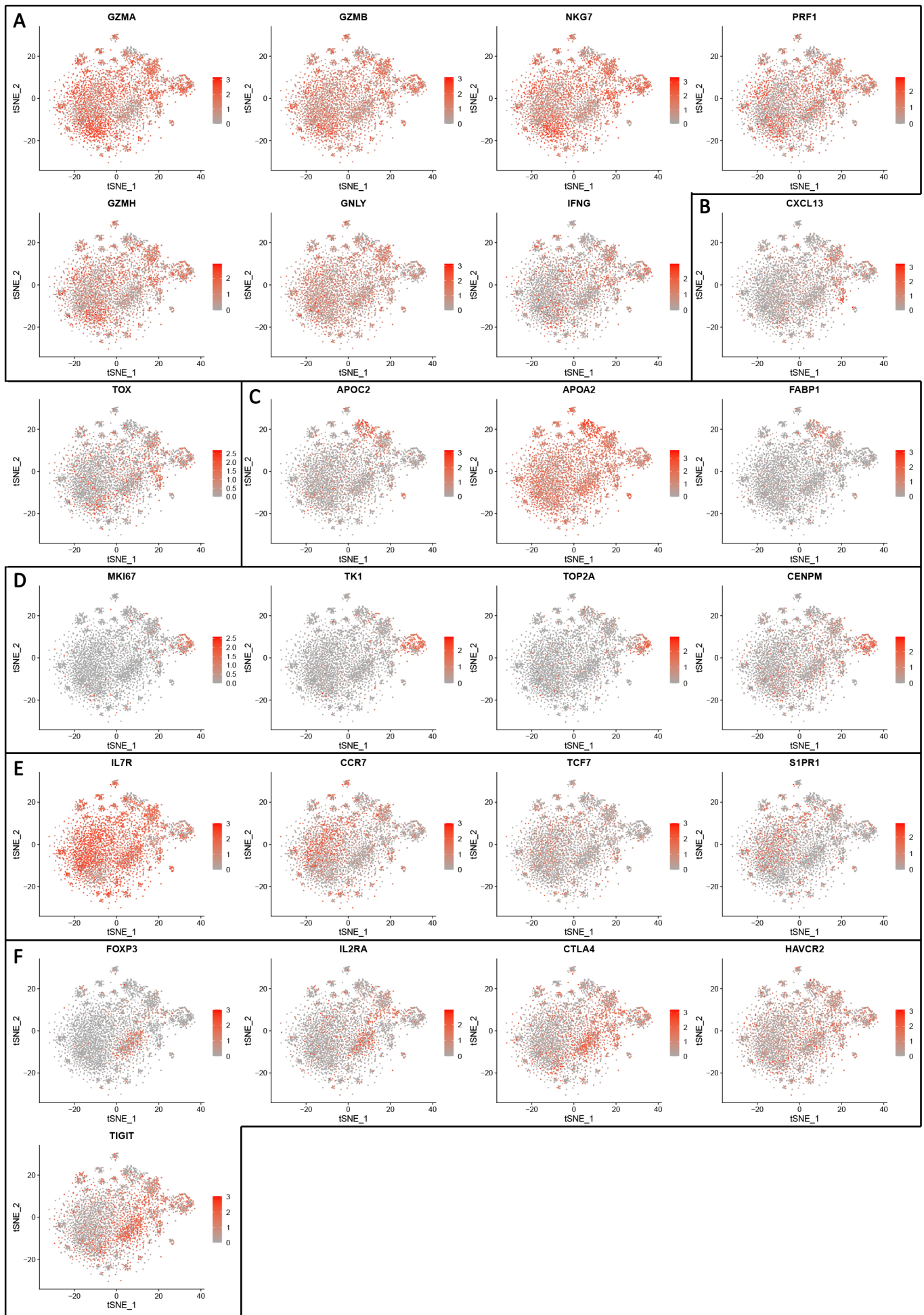

Supplement: Supplementary file 1 [file cells-11-03924-s001.zip › Figure S4 subcluster_supply_merkergene_dimplot.pdf]
